# Characterization of genotype–phenotype correlation with MORC2 mutated Axonal Charcot–Marie–Tooth disease in a cohort of Chinese patients

**DOI:** 10.1186/s13023-021-01881-7

**Published:** 2021-05-31

**Authors:** Xiaohui Duan, Xiaoxuan Liu, Guochun Wang, Weihong Gu, Min Xu, Ying Hao, Mingrui Dong, Qing Sun, Shaojie Sun, Yuanyuan Chen, Wei Wang, Jing Li, Yuting Zhang, Zhenhua Cao, Dongsheng Fan, Renbin Wang, Yuwei Da

**Affiliations:** 1 Department of Neurology, China-Japan Friendship Hospital, Beijing, 100029 People’s Republic of China; 2 Department of Neurology, Peking University Third Hospital, Beijing, 100191 People’s Republic of China; 3 Department of Rheumatology and Immunology, China-Japan Friendship Hospital, Beijing, 100029 People’s Republic of China; 4 Department of Neurology, Xuanwu Hospital, Capital Medical University, Chang Chun Street, Beijing, 100053 People’s Republic of China; 5 Department of Clinical Research Institute, China-Japan Friendship Hospital, Beijing, 100029 People’s Republic of China; 6 Running Gene Inc., Beijing, 100191 People’s Republic of China

**Keywords:** Charcot–Marie–Tooth disease, Spinal muscular atrophy, MORC2, Genotype, Phenotype, Whole-exome sequencing

## Abstract

**Background:**

Charcot–Marie–Tooth (CMT) disease is an exciting field of study, with a growing number of causal genes and an expanding phenotypic spectrum. The microrchidia family CW-type zinc finger 2 gene (MORC2) was newly identified as a causative gene of CMT2Z in 2016. We aimed to describe the phenotypic-genetic spectrum of MORC2-related diseases in the Chinese population.

**Methods:**

With the use of Sanger sequencing and Next Generation Sequencing (NGS) technologies, we screened a cohort of 284 unrelated Chinese CMT2 families. Pathogenicity assessments of MORC2 variants were interpreted according to the ACMG guidelines. Potential pathogenic variants were confirmed by Sanger sequencing.

**Results:**

We identified 4 different heterozygous MORC2 mutations in four unrelated families, accounting for 1.4% (4/284). A novel mutation c.1397A>G p. D466G was detected in family 1 and all affected patients presented with later onset axonal CMT with hyperCKemia. The patient in family 2 showed a spinal muscular atrophy (SMA)-like disease with cerebellar hypoplasia and mental retardation, with a hot spot de novo mutation c.260C>T p. S87L. The twin sisters in family 3 were identified as having the most common mutation c.754C>T p. R252W and suffered from axonal motor neuropathy with high variability in disease severity and duration. The patient in family 4 developed an early onset axonal motor and sensory neuropathy, with a reported mutation c.1220G>A p.C407Y. All identified mutations associated with MORC2-related neuropathies are localized in the N-terminal ATPase module.

**Conclusions:**

Our study confirmed that MORC2-related neuropathies exist in the Chinese population at a relatively high mutation rate. We revealed a complex genotype–phenotype correlation with MORC2 mutations. This report adds a new piece to the puzzle of the genetics of CMT and contributes to a better understanding of the disease mechanisms.

## Background

Charcot–Marie–Tooth (CMT) disease, the most common inherited neurological disease, is a genetically and clinically heterogeneous group of disorders causing progressive degeneration of peripheral motor and sensory neurons. The number of CMT-associated genes has expanded rapidly over the past few decades due to the development of next generation sequencing [1], and more than 80 genes have been linked to different forms of CMT (Neuromuscular Disease Center; http://neuromuscular.wustl.edu/time/hmsn.html).

In 2016, the microrchidia family CW-type zinc finger 2 gene (MORC2) was identified as a causative gene of autosomal dominant axonal Charcot–Marie–Tooth disease type 2Z (CMT2Z, MIM# 616688) in Spanish families [2]. MORC2 is a member of the MORC protein family (MORC1 to MORC4) in human, which shares four conserved domains, an N-terminal catalytically active ATPase module, a central CW-type zinc finger (CW-ZF) domain, a C-terminal chromo-like domain, and distinct coiled-coil (CC) domains [3, 4]. MORC2 encodes a DNA-dependent ATPase, which appears to be involved in many biological functions, such as DNA repair, transcriptional regulation, chromatin remodeling, and lipid homeostasis [5, 6]. These functions suggest several putative roles contributing to the development of axonal CMT.

To date, only ten different MORC2 mutations have been reported to be associated with CMT2Z and related diseases in the Human Gene Mutation Database (HGMD), covering 33 families from Spain, Australia, Czech Republic, Germany, Japan, South Korea and China [7–16]. All of the identified pathogenic variants were located in the GHL-ATPase domain or between the first coiled coil domain and the CW-type zinc finger domain. This new form of axonal CMT presents a number of both early and late onset heterogeneous clinical features, including distal and proximal weakness in an asymmetric and random manner associated with important sensory loss, axonal neuropathy with pyramidal signs, a spinal muscular atrophy (SMA) phenotype, and the appearance of cerebellar atrophy and diaphragmatic paralysis.

The phenotype-genotype correlation in axonal CMT patients with MORC2 mutations is poorly understood because of the complex heterogeneity. In the present study, we performed whole-exome sequencing in a cohort of Chinese patients with unexplained axonal CMT. We also reviewed the literature to further refine the clinical spectrum of MORC2-related neuropathies, to investigate the relationship between the genotype and the phenotype.

## Subjects and methods

### Patients and evaluation

In this study, we enrolled 356 patients from 284 unrelated Chinese CMT2 families. All the patients were enrolled at the Neurology department of China-Japan Friendship Hospital, Peking University Third Hospital and Xuanwu Hospital from 2006 to 2020. The patients underwent a complete neurological examination by two neurologists and met the CMT2 diagnosis criteria based on the clinical phenotype, mode of inheritance, electrophysiologic findings and molecular analyses (PMP22 duplication was excluded) [17, 18]. The study was approved by the respective institutional board of the Ethics Committees of three participating hospitals. Written informed consent was obtained from the patients or their parents involved in this study. They consented to the publication of clinical photographs.

### Genetic analysis

Genomic DNA was isolated from peripheral blood obtained from index patients, their family members and healthy controls using standard procedures. Index patients (n = 146) enrolled from January 2006 to December 2015 were screened by Sanger sequencing and targeted panel sequencing (covering 135 genes, MORC2 was not included in), less than 40% achieved molecular diagnosis. The remaining genetically unidentified index patients (n = 87) and patients (n = 138) enrolled from 2016 to 2020 were screened by whole exome sequencing. The 225 index patients were screened for MORC2 mutations by the following next-generation sequencing methods. Genomic DNA was fragmented into 200–250 bp fragments with the use of sonication. The DNA fragments were sequenced with 150 bp paired-end reads on Illumina HiSeq X10 platform (Illumina, San Diego, USA). Raw data was filtered and aligned against the human reference genome (UCSC hg19) using the Burrows-Wheeler Alignment tool (BWA-0.7.12, http://bio-bwa.sourceforge.net/). Duplicate reads were filtered by Picard, and the single-nucleotide polymorphisms (SNPs), insertions and deletions (indels) were then called by GATK software (www.broadinstitute.org/gatk). All variants in MORC2 were annotated by ANNOVAR (http://annovar.openbioinformatics.org/en/latest/). We confirmed the previously reported pathogenic mutations using Human Gene Mutation Database (http://www.biobase-international.com/product/hgmd), and checked variants against dbSNP (https://www.ncbi.nlm.nih.gov/SNP), 1000Genome (http://browser.1000genome.org), Exome Sequencing Project (http://evs.gs.washington.edu/), Exome Aggregation Consortium (http://exac.broadinstitute.org/) and In-house variation database (Running Gene Inc).

### Sanger sequencing and pathogenicity prediction

The candidate causal variants with clinical significance identified via WES were validated using Sanger sequencing. Co-segregation analyses were conducted with samples from other family members. The effect of single-nucleotide variants (SNVs) was predicted by SIFT (http://sift.jcvi.org/), PolyPhen-2 (http://genetics.bwh.harvard.edu/pph2) and Mutation Taster programs (http://www.Mutationtaster.org). Conservation of the variants among different species was analyzed using BioEdit Sequence Alignment Editor (North Carolina St. University, USA) to align with the reference sequences in Ensemble database (http//useast.Ensemble.org/index.html). Pathogenicity of identified variants was assessed according to the standards and guidelines of American College of Medical Genetics and Genomics (ACMG) [19].

### Electrophysiological studies

The electrophysiological studies were performed using a Dantec Keypoint machine with standard methods, skin temperature was maintained in the range of 32–34 °C. Nerve conduction studies and needle electromyography (EMG) studies were analyzed in three affected members (proband IV-9, patient V-2, V-3) from family 1, and all patients from other families.

### Paraclinical investigations

Sural nerve and musculus biceps brachii biopsies from proband IV-9 in family 1 and proband in family 2 were analyzed using light and electron microscopy (Nikon type 108,Japan). Skeletal muscle and nerve MRIs were performed on a 3.0 T machine (Siemens, Germany) using an anatomic 1.5 mm isotropic Cube-STIR-T2WI sequence in proband IV-9 and patient V-3 of family 1. Blood laboratory tests were performed in all patients from four families and some unaffected families’ members.

## Results

### Genetic findings and analysis

**Fig. 1:**
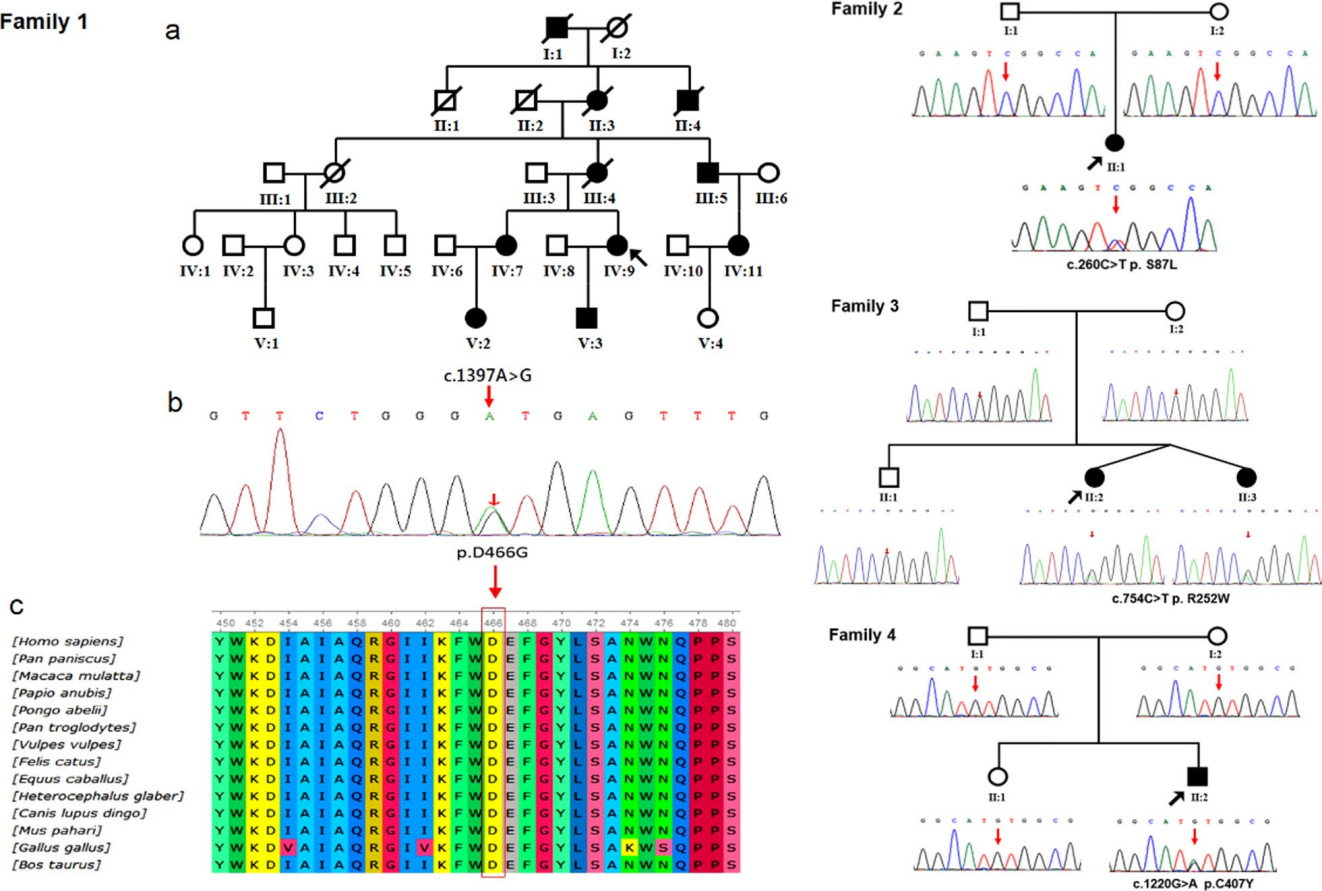
(Left) A novel mutation in family 1. **a**: pedigree of family 1 showed 10 affected and 8 unaffected members over five generations. **b**: chromatograms showed sequencing analysis of the novel heterozygous mutation c.1397A>G p. D466G. **c**: the affected amino acid is highly conserved among different species. (Right) Chromatograms sequencing analysis of the related de novo heterozygous mutations in family 2, 3 and 4 pedigrees. Circles: females; squares: males; shaded symbols: affected individuals; arrows: probands; /: dead individuals

Among the 284 patients, 4 different heterozygous mutations were detected by whole exome sequencing and segregated with the MORC2-related disease in four unrelated families. We summarize the pedigrees and genotypes of families with MORC2 mutations in this study (Fig. 1).

In family 1, a novel mutation c.1397A>G p. D466G was identified. The pathogenicity of the novel missense mutation is supported by the following evidences: (i) the mutation perfectly segregated with the disease (it was identified in all six affected patients and was absent in four healthy members of the family); (ii) variations in amino acid at the same site have been identified as pathogenic; (iii) the variant is not present in the ExAC database, 1000 Genomes project and Genome Aggregation Database, and which is not found in Human Gene Mutation Database; (iv) the affected amino acid is highly conserved among different species; and (v) the in silico prediction tool SIFT, PolyPhen-2, and Mutation Taster all define it as damaging (1.0) or probably damaging (0.997). According to ACMG guidelines, the novel c.1397A>G p. D466G should be categorized as a “likely pathogenic variant”because it belongs to PM1, PM2, PM5, PP1, PP3 as well as PP4.

In family 2, a de novo mutation c.260C>T p. S87L was identified. It has been reported to cause spinal muscular atrophy-like phenotype in one Spanish and two Koreans [2, 10]. Considering the different ethnicities of these patients, p.S87L was defined as mutational hot spot.

In family 3, a de novo mutation c.754C>T p.R252W was confirmed with Sanger sequence in the twin sisters but not in their parents or brother. This was the most common mutation which was reported in other countries [2, 7–9, 15]. In this study, we revealed it in Chinese population for the first time.

In family 4, the mutation c.1220G>A p.C407Y was identified in the patient but not in his parents or sister, indicating the de novo origin of the mutation, similarly to a reported Japanese patient [15].

### Clinical features

#### Family 1

The family included 10 affected and 8 unaffected members over five generations. The proband (IV-9), a 46-year-old woman, presented with progressive weakness of upper and lower limbs for 10 years. At the age of 36, she noticed numbness and coldness in both hands. Then she had difficulty in doing housework because of hand weakness and muscle atrophy. Sensation in both hands decreased, albeit more so in the left hand. It is hard for her to wring out a towel, to write, and even to use chopsticks. The patient’s conditions worsened as time went by. At 40 years of age, she started to have trouble with walking and lost sensation in her feet. Cold would aggravate the symptoms and occasionally she felt tremors in hands. Neurological examination at 46 years old revealed prominent muscle atrophy and weakness in the distal legs and hands. The foot and ankle flexor–extensor muscles strength scored 4-/5 on the Medical Research Council (MRC) scale, the finger abductor and adductor muscles strength were 4/5 on MRC. There was moderate sensory reduction of all modalities in the distal regions below knees or elbows. Deep tendon reflexes were diminished in the upper limbs and absent in the lower limbs. Pyramidal tract signs were not observed. Steppage gait and flat feet were present. Serum CK level (assessed after rest) was elevated at 1037 IU/L (normal range, 25–200 IU/L), while other laboratory studies were normal, including complete blood count, erythrocyte sedimentation rate, C-reactive protein, serum biochemical indexes, antinuclear antibody (ANA), anti-DNA antibody, antineutrophil cytoplasmic antibodies (ANCA), circulating C3 and C4 et cetera.

Patient V-3 (the proband’s 19-year-old son) did not complain of paresthesia or weakness of his limbs. At neurological examination, he presented slight muscles atrophy and weakness (4+/5 on MRC) in both hands, weakened tendon reflexes in the distal limbs. Decreased sensation was observed in glove-stock distribution. Serum CK level (assessed after rest) was also elevated at 495 IU/L.

Other affected individuals all presented similar symptoms with numbness and weakness in the distal limbs. Hand weakness appeared before that of distal lower limbs in most patients. At the most recent examination, all surviving patients showed a common sign characterized by weakness and atrophy, reduced or absent deep tendon reflexes and sensory deficit in the distal limbs. Walking abilities were limited or reduced in all, patient III-5 was wheelchair dependent, others maintained the ability to walk without assistance. The phenotype severity was assessed by the CMTNS (range 4–32) (Table 1).

**Table 1:** Summarizes the characteristics of genotype and phenotype in all patients from three families

Patients ID	Nucleotide change	AA change	Sex	Age (y) at Onset	Age (y) at Exam	F H	Initial symptoms	Clinical features	Proximal involvement	Laterality	Muscle strength		Sensory loss		Tendon reflex		CMTNS	Additional features
											UL	LL	UL	LL	UL	LL		
Family 1 IV 9	c.1397A>G	p.D466G	F	36	46	AD	Numbness in hands	Hands weakness and atrophy, Gait difficulties, Distal dysaesthesia, Foot drop	No	Left	4-/D	4/D	P, T, V/D	P, T, V/D	Decreased	Absent	18	Hands tremor hyperCKemia (1037 IU/L)
Family 1 III 5			M	30	68		Weakness in hands	Weakness and atrophy in distal limbs, Distal dysaesthesia, Pes cavus, Foot drop	Yes	–	4/P; 3/D	3/P; 2/D	P, T, V/D	P, T, V/D	Absent	Absent	32	hyperCKemia (958 IU/L)
Family 1 IV 7			F	27	51		Weakness in hands	Weakness and atrophy in distal limbs, Distal dysaesthesia, Pes cavus, Foot drop	Yes	Left	4+/P; 4-/D	4+/P; 4-/D	P, T, V/D	P, T, V/D	Decreased	Absent	29	Hands tremor hyperCKemia (869 IU/L)
Family 1 IV 11			F	28	38		Numbness in hands	Weakness and atrophy in distal limbs, Distal dysaesthesia, Foot drop	No	–	4/D	4/D	P, T, V/D	P, T, V/D	Decreased	Absent	25	Hands tremor hyperCKemia (736 IU/L)
Family 1 V 2			F	25	27		Hands tremor	Hands weakness and atrophy, Gait difficulties, Distal dysaesthesia	No	–	4/D	4/D	P, T, V/D	P, T, V/D	Decreased	Decreased	14	HyperCKemia (1639 IU/L)
Family 1 V 3			M	19	19		–	Slight weakness and atrophy in both hands	No	–	4+/D	4+/D	P, T/D	P, T/D	Decreased	Decreased	4	HyperCKemia (495 IU/L)
Family 2 II 1	c.260C>T	p.S87L	F	< 1	7	SF	Motor develop delay	Generalized weakness and atrophy, Dysarthria Scoliosis, Claw hand, Flat feet	Yes	–	3/P; 4/D	2/P; 2/D	P, T, V/D	P, T, V/D	Absent	Absent	31	Cerebellar hypoplasia, mental retardation
Family 3 II 2	c.754C>T	p.R252W	F	< 1	10	SF	Motor develop delay	Weakness in distal lower limbs, Steppage gait, Pes cavus, Strephenopodia	No	–	5/D	4/D	P, T/D	P, T/D	Decreased	Absent	10	–
Family 3 II 3			F	6	10	SF	Foot deformity	Mild gait disturbance, Pes cavus, Strephenopodia	No	–	5/D	4+/D	–	–	Decreased	Decreased	2	–
Family 4 II 2	c.1220G>A	p.C407Y	M	6	19	SF	Motor difficulty	Weakness and atrophy in distal limbs, Distal dysaesthesia, Pes cavus, Hammer toes	No	–	4/D	3+/P; 2/D	P, T/D	P, T/D	Decreased	Decreased	20	–

F = Female; M = Male; CMTNS = Charcot–Marie–Tooth neuropathy score; FH = Family History; AD = autosomal dominant inheritance; SF = Sporadic families; UL = Upper limbs; LL = Lower limbs; P = Proximal; D = Distal; P = pinprick; V = vibratory; T = Touch

Electrophysiological studies (Table 2) showed axonal motor and sensory neuropathy in proband IV-9 and patient V-2, and mild sensory axonal peripheral neuropathy in patient V-3.Needle EMG revealed chronic neurogenic changes, spontaneous muscle activity or satellite potential.

**Table 2:** Electrophysiological features in affected members of three families

Nerve	Family 1-IV9			Family 1-V2			Family 1-V3			Family 2 II-1			Family 3 II-2			Family 3 II-3			Family 4 II-2		
	MCV, m/s	DL, ms	CMAP, mV	MCV, m/s	DL, ms	CMAP, mV	MCV, m/s	DL, ms	CMAP, mV	MCV, m/s	DL, ms	CMAP, mV	MCV, m/s	DL, ms	CMAP, mV	MCV, m/s	DL, ms	CMAP, mV	MCV, m/s	DL, ms	CMAP, mV
*Median*																					
E-W	62.50	6.36	3.50↓	55.30	6.42	4.00↓	56.10	6.64	13.50	42.10	4.87	0.12↓	48.80	7.10	10.00	48.80	7.40	10.50	50.10	9.90	1.16↓
W-APB		3.24	4.90↓		2.68	6.71↓		2.81	14.20		3.09	0.37↓		2.90	11.50		3.20	10.40		5.71↑	1.22↓
*Ulnar*																					
E-W	63.60	5.48	17.00	58.10	5.84	11.60	57.10	6.20	13.40	52.90	5.90	0.10↓	50.00	6.30	7.30	42.50	6.30	7.60	42.90	7.57	0.38↓
W-ADM		2.57	17.20		2.57	13.10		2.52	14.00		2.50	0.10↓		2.00	7.90		1.83	8.60		3.96↑	0.50↓
*Tibial*																					
K-A	49.40	10.80	7.00	43.40	11.70	2.60↓	53.10	11.40	13.90	35.10↓	9.40	0.30↓	41.30	10.20	8.50	39.70	11.40	8.20	36.50↓	15.10	1.37↓
A-FHB		3.10	10.30		2.83	3.30↓		3.96	13.60		3.70	0.20↓		2.70	10.80		2.70	11.30		4.68	2.10↓
*Peroneal*																					
K-A	48.10	8.84	1.22↓	36.90↓	12.60	0.10↓	56.00	10.40	10.30	40.00↓	6.80	0.10↓	42.10	14.90	0.30↓	46.40	11.50	1.10↓	NR	NR	NR
A-EDB		2.81	1.31↓		4.75	0.13↓		3.45	12.90		2.80	0.14↓		7.30	0.20↓		4.40	1.30↓		NR	NR

	SCV, m/s	DL, ms	SNAP, µV	SCV, m/s	DL, ms	SNAP, µV	SCV, m/s	DL, ms	SNAP, µV	SCV, m/s	DL, ms	SNAP, µV	SCV, m/s	DL, ms	SNAP, µV	SCV, m/s	DL, ms	SNAP, µV	SCV, m/s	DL, ms	SNAP, µV
*Median*																					
Dig I-W	NR	NR	NR	37.00↓	3.84	1.55↓	45.60↓	2.54	1.12↓	NR	NR	NR	50.00	2.00	8.80	56.20	1.69	11.00	NR	NR	NR
*Ulnar*																					
Dig V-W	NR	NR	NR	30.70↓	3.26	1.34↓	45.90↓	2.66	0.63↓	NR	NR	NR	ND	ND	ND	ND	ND	ND	NR	NR	NR
*Peroneus*																					
Dig I-Ankle	NR	NR	NR	NR	NR	NR	34.40↓	4.88	0.52↓	NR	NR	NR	41.40	1.69	9.30	45.80	1.42	5.20	NR	NR	NR
*Needle EMG*																					
Abductor pollicis brevis	Chronic neurogenic			Chronic neurogenic Spontaneous muscle activity			Chronic neurogenic			Chronic neurogenic Spontaneous muscle activity			ND			ND			ND		
Musculus biceps brachii	Chronic neurogenic			Chronic neurogenic Spontaneous muscle activity			Chronic neurogenic			Chronic neurogenic Spontaneous muscle activity			ND			ND			ND		
Abductor digiti minimi	Satellite potential			ND			ND			ND			ND			ND			ND		
Tibialis anterior	Chronic neurogenic			Chronic neurogenic Spontaneous muscle activity			Chronic neurogenic			Chronic neurogenic Spontaneous muscle activity			ND			ND			ND		
Quadriceps	Chronic neurogenic			Chronic neurogenic Spontaneous muscle activity			ND			Chronic neurogenic Spontaneous muscle activity			ND			ND			ND		

A = ankle; ADM = abductor digiti minimi; APB = abductor pollicis brevis; CMAP = compound muscle action potential; DL = distal latency; Dig = digit; E = elbow; EDB = extensor digitorum brevis; FHB = flexor hallucis brevis; K = knee; MCV = motor conduction velocity; SCV = sensory conduction velocity; SNAP = sensory nerve action potential; W = wrist; ND = not done; NR = no response

**Fig. 2:**
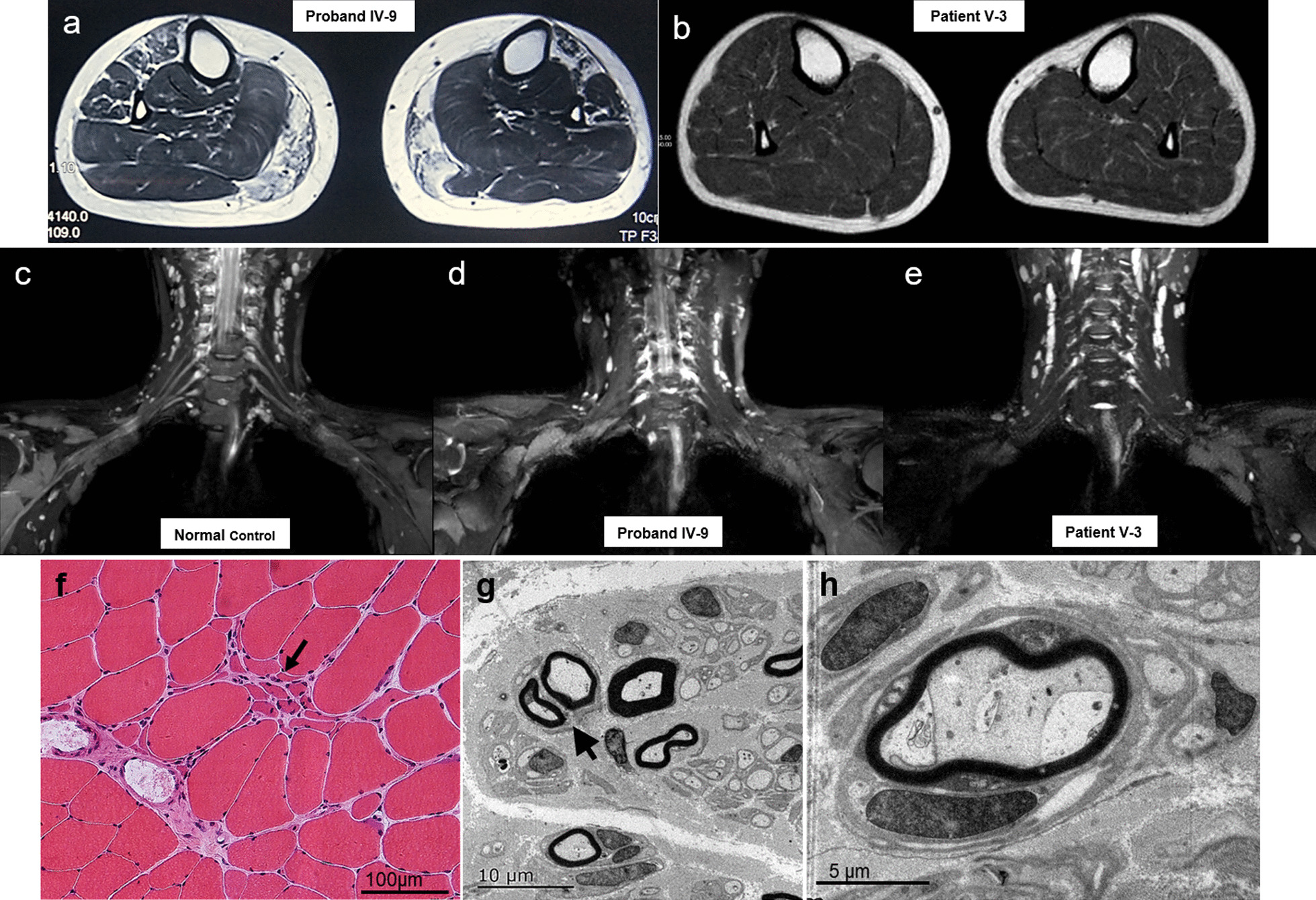
Imaging and neuropathological features of family 1. MRI of calf skeletal muscles (Axial T2W1-STIR) in proband IV-9 exhibited slight fatty infiltration in the bilateral anterior tibial muscle and extensor hallucis longus, and distinct fatty infiltration in the gastrocnemius muscles (**a**). Her son V-3 displayed almost normal muscle morphology (**b**). MRI of nerves (Coronal 3D-STIR) showed atrophy of cervical nerve roots and brachial plexus in proband IV-9 (**d**) and patient V-3 (**e**). Musculus hematein eosin section of proband IV-9 revealed neurogenic abnormalities with small angular atrophic muscle fibers distributed in clusters (**f**, black arrow). Electron microscope micrographs of sural nerve showed a regenerative cluster composed of small myelinated fibers (**g**, black arrow) and occasional atypical onion bulb (**h**)

MRI of calf skeletal muscles in proband IV-9 exhibited multiple abnormal signals in bilateral calf muscles, fatty infiltration of the bilateral anterior tibial muscle, extensor hallucis longus and gastrocnemius muscles. Her son V-3 displayed almost normal muscle morphology (Fig. 2a, b). MRI from nerve roots to nerve trunks in the upper arms showed atrophy of cervical nerve roots and brachial plexus in proband IV-9 and patient V-3 (Fig. 2c–e).

Proband IV-9 musculus biceps brachii biopsies showed neurogenic abnormalities with small angular atrophic muscle fibers distributed in clusters (Fig. 2f). Sural nerve biopsy revealed pronounced multifocal depletion of large myelinated fibers with some regenerative clusters and occasional atypical onion bulbs (Fig. 2g, h).

#### Family 2

**Fig. 3:**
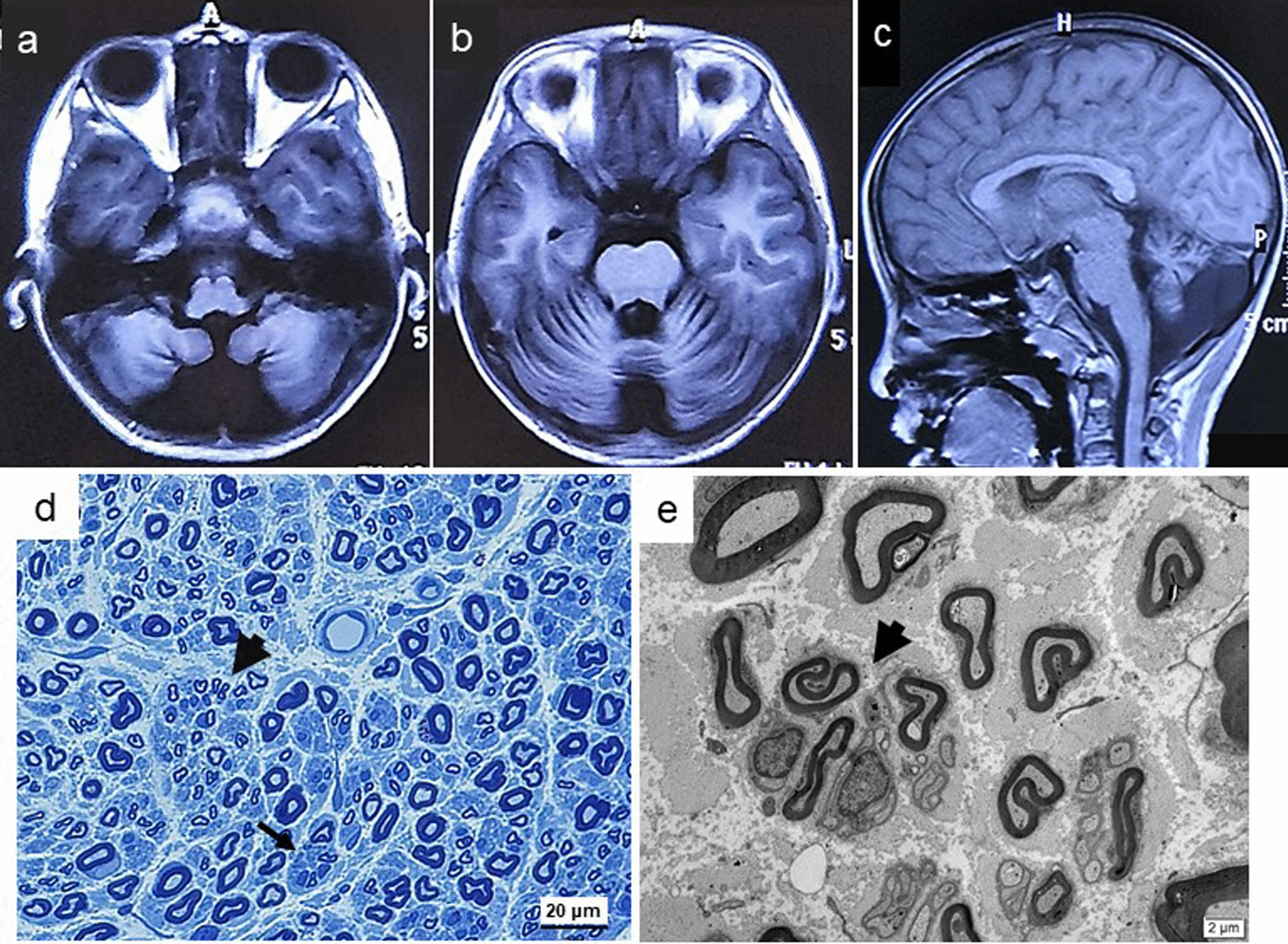
Imaging and neuropathological features of family 2. T1-weighted axial and mid-sagittal in Brain MRI showed a large occipital cistern cyst and severe hypoplasia of the cerebellum (**a**–**c**). Transverse semi-thin section of sural nerve revealed multifocal loss of large myelinated nerve fibers with increased thin myelinated fibers; regenerative clusters (black arrowhead) and axonal degeneration (thin arrow) were seen (**d**). Electron microscope micrographs in a low power view showed a regenerative cluster (**e**, black arrowhead)

The index patient was a 7-year-old girl, who was born from healthy non consanguineous Chinese parents, there is no family history of genetic disease. She presented with a delay in the acquisition of motor and mental milestones after a normal pregnancy and delivery. She achieved turn over body but raised head unsteadily at 8 months. She achieved passive sedestation but neither independent bipedestation nor crawling at 16 months. At two and a half years, she achieved active sedestation and bipedestation but not independent ambulation. When 7 years old, she was admitted to hospital in a wheelchair. She was unable to walk independently and needed a walker. At neurological examination, we recognized her stature and intelligence were developmentally delayed. Minor facial anomalies such as short forehead, anteverted nares, flat philtrum, thick lips, and micrognathia were also present. Dysarthria was noticed since milestones of communicating. The patient showed generalized weakness including slight facial and lip involvement, generalized muscular hypotonia and areflexia. Muscular weakness in the upper limbs (3 to 4/5 on MRC) and lower limbs (2/5 on MRC). Scoliosis, claw hands, and flat feet were observed. Sensitive impairment was mild, characterized by hypoaesthesia in distal limbs. No pyramidal signs were observed. Electrophysiological study (at 7 years old) showed axonal motor and sensory neuropathy (Table 2). Brain MRI at 7 years showed large occipital cistern cyst and severe hypoplasia of the cerebellum (Fig. 3a–c). Sural nerve biopsy of the patient revealed multifocal loss of large myelinated nerve fibers with increased thin myelinated fibers; regenerative clusters and axonal degeneration were seen (Fig. 3d, e).

#### Family 3

The twin sisters were 10-year-old, who was born at 35 weeks of gestation from healthy non consanguineous Chinese parents. Their parents and an older brother are reportedly healthy. There is no family history of genetic disease. One twin sister showed a delay in the acquisition of motor milestones. She started walking independently at 2 years old. She was unable to run and jump like the same aged children. She developed gait disturbance (steppage gait), foot deformity (pes cavus, strephenopodia) and distal muscular atrophy at 4 years old. Her twin sister reached early developmental milestones normally. Her first signs were foot deformity (pes cavus, strephenopodia) and a slightly abnormal gait at the age of 6. She never complained about difficulty with walking, running or jumping. Neurological examination at age 10 years, the twin sisters presented with normally development in stature and intelligence. Muscular strength in the distal lower limbs was 4/5 on MRC in the early onset sister, and 4+/5 on MRC in the late onset sister. Sensitive impairment was mild in distal limbs. Tendon reflexes were reduced or absent. No pyramidal signs were observed. Electrophysiological studies (at 10 years old) in twin sisters both revealed axonal motor neuropathy (Table 2).

#### Family 4

The now 19-year-old patient was born at term to healthy non-consanguineous Chinese parents. An elder sister is healthy, there is no family history of genetic disease. He described motor difficulties at 6 years of age (always walked and ran slower than the same aged children, but without a delay in achieving walking). The disease rapidly progressed, and at age 12, a distinct weakness and atrophy in the distal lower limbs muscles were observed. At age 17 years he developed numbness and weakness in the distal upper limbs. At that, he could only walk a maximum distance of 800 m without assistance and only climb stairs with a banister, and he was unable to squat down or stand up. Moreover, he reported having poor handwriting and difficulties in using zippers and buttons. Neurological examination at age 17 years showed muscle atrophy and weakness in the distal limbs (upper limbs 4/5 on MRC, lower limbs 2 to 3/5 on MRC). Foot deformity, hammer toes and calluses on the external side of the feet were noticed. Atrophies of thenar and hypothenar muscles, and forearm muscles were observed. Decreased sensation was observed in glove-stock distribution as well as weakened tendon reflexes in the distal limbs. There were no pyramidal signs. Electrophysiological study (at 19 years old) showed axonal motor and sensory neuropathy (Table 2).

## Discussion

The field of CMT and related diseases has developed rapidly in recent years, with the discovery of a growing number of causal genes and an expanding phenotypic spectrum. MORC2-related axonal CMT disease was first described in 2016 as later onset CMT2 or early onset spinal muscular atrophy-like phenotype [2]. Recent studies on MORC2 mutations show a remarkable heterogeneity in clinical features and disease severity, ranging from axonal motor and sensory neuropathy to a complex multisystem disorder.

In this study, we identified 4 index patients in unrelated Chinese families with MORC2 mutations, accounting for 1.4% (4/284) in our cohort. A recently published study in north China has reported a MORC2 mutation frequency rate of 1.7% (2/115) [10]. Combining the data on the prevalence rate as identified in previous studies in Korea 2.6% (4/152) [9], Japan 2.7% (13/487) [15] and the present study, we find that the mean frequency distribution rate of MORC2 mutations in the East Asian population is 2.2% (23/1038). Our study confirmed that MORC2 is a CMT2 disease-causing gene of relatively high mutation rate. Of interest, the MORC2 mutations have shown a high rate of de novo events. In our four families, de novo mutations were confirmed in three families by testing the parents.

Reviewing previous literature, patients carrying MORC2 mutations showed a wide and complex phenotypic spectrum: (i) late onset or early onset axonal CMT [2, 8, 10, 13, 15]; (ii) congenital or early onset SMA-like syndrome [2, 9, 13, 16]; (iii) axonal neuropathy plus CNS symptoms (pyramidal signs, seizures, leukomalacia, mental retardation, spinal cord atrophy, tremor, hearing loss, et al.) [7, 9, 15]; (iiii) axonal neuropathy plus multisystemic disorders (cerebellar atrophy, diaphragmatic paralysis, nocturnal hypoventilation, scoliosis, dysmorphic face, et al.) [12, 14]. These findings support a new and more comprehensive recognition of MORC2-related diseases as a multisystemic spectrum.

Our cohort study provided new evidence supporting this notion. Patients belonging to Family 1 showed an adult onset of chronic axonal motor and sensory neuropathy associated with hyperCKemia. The most frequent initial symptom was hand weakness, and the common clinical feature was distal limbs weakness and sensory loss. Previous reported clinical characteristics, proximal limbs involvement, prominent sensory disturbances and asymmetric impairment [2, 10] also presented in some patients within this family. All affected individuals had another uncommon clinical manifestation—hyperCKemia. Elevated CK concentration has been reported in axonal CMT with MPZ and NEFL mutations [20–23], but the cause of hyperCKemia remains unclear. Possible mechanisms could include impaired muscle membrane integrity caused by denervation deriving from axonal damage [20], or an altered or slowed muscle fiber type differentiation caused by inadequate nerve function [23]. In this family, the presence of spontaneous muscle activity at EMG (proband 1-IV9, patient 1-V2, patient 1-V3) and neuropathic changes in muscle biopsy (proband 1-IV9) confirmed active denervation together with chronic neurogenic changes. Other possible genetically causes of myopathy were ruled out, however, the possibility of a coincidental association between an idiopathic hyperCKaemia and MORC2 mutation cannot be excluded.

The patient in Family 2 presented with a delay in the acquisition of motor and mental milestones, then developed to a SMA-like disease with cerebellar hypoplasia and mental retardation. All reported patients (one Spanish and two Koreans) with the p.S87L mutation exhibited similar SMA-like phenotype [2, 9]. Schottmann [12] and Zanni [14] each respectively described a German patient and an Italian patient with de novo p.T424R mutation associated with SMA-like neuropathy, cerebellar atrophy, diaphragmatic paralysis or nocturnal hypoventilation. Our report confirmed that patients carrying the p.S87L or p.T424R mutation shared a similar phenotype of SMA-like disease with complex syndrome. Sancho observed that the p.S87L mutation of MORC2 led to an increase in axonal swelling in neurons, which represented abnormal accumulation of axonal cargos and cytoskeletal proteins, directly affected the axonal transport systems through microtubules and motor proteins [24]. As a hallmark of axonal injury, axonal swelling underlie the pathogenesis of the neuropathy and contribute to disease severity and progression.

The twin sisters in Family 3 suffered from axonal motor neuropathy with high variability in disease severity and duration. One twin sister presented disease onset in early infancy with delays in the acquisition of motor milestones, never walked normally and never ran or jumped. A rapid progression was further noticed in foot deformity and distal muscular atrophy. The other twin sister described the first signs of foot deformity and slightly abnormal gait at 6 years of age, without any difficulty with walking, running and jumping. It is speculated that the phenotypic differentiation of twins may be caused by epigenetic modification, such as DNA methylation, histone modification, and microRNAs-mediated regulation. Mechanisms of epigenetics regulate gene expression and display some level of phenotypic discordance.

The patient of Family 4 showed a typical axonal motor and sensory neuropathy, developed early onset (first decade) and rapid progression. A reported Japanese patient with the same mutation showed almost the same phenotypes as follows: the age at onset, the most frequent initial symptom as cramps in the lower limbs, distal lower limb weakness and sensory loss during the initial examination [15].

**Fig. 4:**
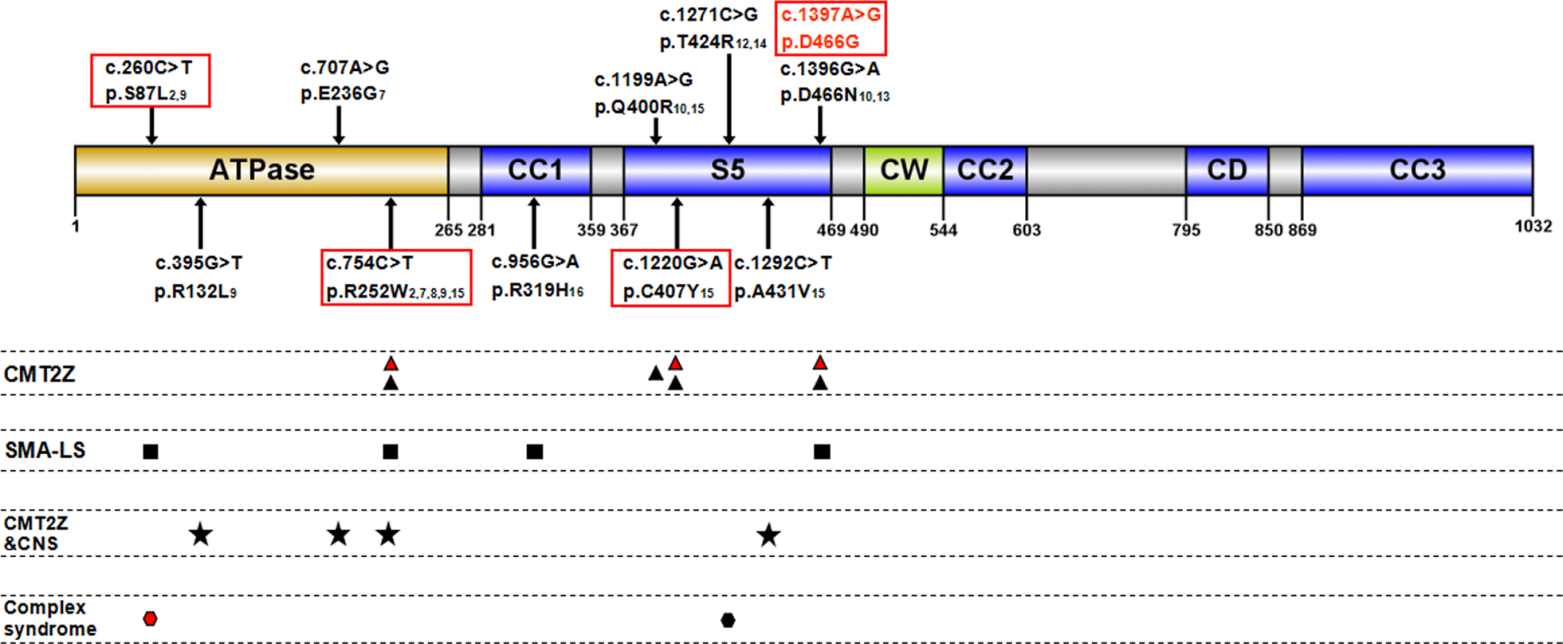
Graphical overview of MORC2 protein structure with the localization of mutations identified in CMT2Z patients. ATPase: GHL (Gyrase B, Hsp90, and MutL)-ATPase domain at the amino-terminus; CC1, 2 and 3: coiled-coil domains; S5: ribosomal protein S5 domain; CW: CW-type finger domain; CD: Chromo- like domain. Red boxes: mutations were identified in this study, red words indicated a novel mutation. Black triangles: mutations showed late onset or early onset axonal CMT; Black squares: mutations showed congenital or early onset SMA-like syndromes; Black pentacles: axonal neuropathy plus CNS symptoms; Black hexagons: axonal neuropathy plus multisystemic disorders; Red triangles and red hexagon: phenotypes were described in this study

Genotype–phenotype correlation is still unclear in MORC2-mutated patients. To explore the correlation between genotype and phenotype, we summarized all reported patients with MORC2-mutated phenotype (Fig. 4).

MORC2 is a 1032-amino acid (AA) protein predicted to contain several functional domains. The N-terminal catalytically active ATPase module is composed of Gyrase B, Hsp90, histidine kinase, and MutL (GHKL) and S5-fold domains (AA residues 1–469), which has been mechanistically linked to gene transcription and DNA repair by remodeling chromatin [3, 4]. The CW-type zinc finger domain (AA residues 490–544) participates in chromatin regulation through the recognition of epigenetic signals [4]. The C-terminal chromo-like domain (AA residues 795–850) is commonly found in eukaryotic chromatin proteins and can recognize methylated peptides in histones and nonhistone proteins [25]. The coiled-coil domains are suggested to be important structural determinants for protein assembly and molecular recognition [25]. To date, four MORC2 mutations (p.S87L, p.R132L, p.E236G and p.R252W) are known to be located in the ATPase domain, one MORC2 mutation (p.R319H) is located in the first coiled-coil domain, five other known MORC2 mutations (p.Q400R, p.C407Y, p.T424R, p.A431V and p.D466N) and the novel mutation p. D466G in this study are located in the ribosomal protein S5 domain. All identified mutations associated with MORC2-related neuropathies are localized in the N-terminal ATPase module, which might be critical to MORC2 protein function.

It is interesting to note that the different mutations causing distinct changes in biochemical properties (Table 3) [6], which may be helpful to understand how MORC2 mutations cause the complex range of clinical symptoms. The p.R252W mutation is supposed to be the most common mutation, and with a highly variable clinical features including late onset or early onset axonal CMT, axonal neuropathy plus CNS symptoms, and congenital or early onset SMA-like syndrome [2, 7–9, 15]. Recent study showed R252W mutation hyperactivated HUSH-mediated epigenetic silencing in neuronal cells, which weakened the regulatory ATPase-CW interaction [5]. Another known mutational hotspot is p.D466N, which is associated with axonal CMT and SMA-like syndrome [10, 13]. In our study, we identified a novel mutation p. D466G caused symptom of axonal CMT with hyperCKemia, which provided new evidence. This mutation caused destabilization in ATPase similar to R252W. The other three mutations p.S87L, p.R319H and p.T424R presented with SMA-like syndromes or plus complex disorders [2, 9, 12, 14]. Some research showed S87L caused kinetic stabilization of MORC2 dimers, whereas T424R increased the rate of dimer assembly and disassembly [5]. These two biochemical effects were more distinct and affected patients presenting with severe phenotype of early onset. The p.Q400R and p.C407Y mutations were both shown as typical axonal CMT [10, 15], otherwise p.R132L, p.E236G, p.A431V manifested as axonal neuropathy plus CNS symptoms [7, 9, 15]. The mechanism by which these mutations cause changes in biochemistry is unclear, so more work will be needed to explore how MORC2 mutations cause the complex range of clinical symptoms.

**Table 3:** Summary of the molecular consequences of neuropathic and structure-based mutants of MORC2

Mutation	Activity relative to wild-type MORC2		Position in structure	Proposed mechanism of MORC2 misregulation
	ATPase	HUSH		
S87L	Lower	Higher	ATP lid	Constitutive N-terminal dimerization
R132L	Not determined	Not determined	ATPase core	Destabilize ATPase
E236G	Not determined	Not determined	ATPase core	Destabilize ATPase
R252W	Lower	Higher	ATPase-CW interface	Destabilize ATPase-CW module
Q400R	Not determined	Not determined	ATPase core	Destabilize ATPase
T424R	Higher	Lower	Dimer interface	Perturb dimerization dynamics
D466N	Not determined	Not determined	ATPase surface	Destabilize ATPase

In conclusion, we described four Chinese axonal CMT families caused by MORC2 mutations, accompanied by hyperCKemia, cerebellar hypoplasia and mental retardation. Our findings expand the ethnic, phenotypic, and genetic diversity of MORC2-related neuropathies. It should be considered in the diagnostic workup of unresolved cases of axonal neuropathy, SMA-like syndrome, and with other complex associated phenotypes. MORC2 mutations are transmitted as an autosomal dominant trait, with a high rate of dominant de novo mutations. Further studies may elucidate the mechanism underlying the diversity of MORC2-related diseases.

## Data Availability

The datasets used in this study are available from the corresponding author upon request.
